# Efficacy of Anti-HER2 Agents in Combination With Adjuvant or Neoadjuvant Chemotherapy for Early and Locally Advanced HER2-Positive Breast Cancer Patients: A Network Meta-Analysis

**DOI:** 10.3389/fonc.2018.00156

**Published:** 2018-05-22

**Authors:** Márcio Debiasi, Carisi A. Polanczyk, Patrícia Ziegelmann, Carlos Barrios, Hongyuan Cao, James J. Dignam, Paul Goss, Brittany Bychkovsky, Dianne M. Finkelstein, Rodrigo S. Guindalini, Paulo Filho, Caroline Albuquerque, Tomás Reinert, Evandro de Azambuja, Olufunmilayo Olopade

**Affiliations:** ^1^School of Medicine, Pontifícia Universidade Católica do Rio Grande do Sul, Porto Alegre, Brazil; ^2^National Institute for Health Technology Assessment (IATS), Universidade Federal do Rio Grande do Sul, Porto Alegre, Brazil; ^3^LACOG (Latin American Cooperative Oncology Group), Porto Alegre, Brazil; ^4^Hospital do Câncer Mãe de Deus, Porto Alegre, Brazil; ^5^University of Missouri, Columbia, SC, United States; ^6^Department of Public Health Sciences, The University of Chicago, Chicago, IL, United States; ^7^Harvard Medical School, Boston, MA, United States; ^8^Massachusetts General Hospital, Boston, MA, United States; ^9^Dana-Farber Cancer Institute, Boston, MA, United States; ^10^Department of Radiology and Oncology, The State of Sao Paulo Cancer Institute, University of Sao Paulo Medical School, Sao Paulo, Brazil; ^11^CLION, CAM Group, Salvador, Brazil; ^12^Department of Medical Oncology, Hospital São Lucas da PUCRS, Porto Alegre, Brazil; ^13^Institut Jules Bordet and Université libre de Bruxelles (ULB), Brussels, Belgium; ^14^Center for Innovation in Global Health, Department of Medicine, The University of Chicago, Chicago, IL, United States

**Keywords:** breast cancer, HER2/ERBB2, adjuvant treatment, neoadjuvant treatment, meta-analysis, network meta-analysis

## Abstract

**Background:**

Several (neo)adjuvant treatments for patients with HER2-positive breast cancer have been compared in different randomized clinical trials. Since it is not feasible to conduct adequate pairwise comparative trials of all these therapeutic options, network meta-analysis offers an opportunity for more detailed inference for evidence-based therapy.

**Methods:**

Phase II/III randomized clinical trials comparing two or more different (neo)adjuvant treatments for HER2-positive breast cancer patients were included. Relative treatment effects were pooled in two separate network meta-analyses for overall survival (OS) and disease-free survival (DFS).

**Results:**

17 clinical trials met our eligibility criteria. Two different networks of trials were created based on the availability of the outcomes: OS network (15 trials: 37,837 patients); and DFS network (17 trials: 40,992 patients). Two studies—the ExteNET and the NeoSphere trials—were included only in this DFS network because OS data have not yet been reported. The concept of the dual anti-HER2 blockade proved to be the best option in terms of OS and DFS. Chemotherapy (CT) plus trastuzumab (T) and lapatinib (L) and CT + T + Pertuzumab (P) are probably the best treatment options in terms of OS, with 62.47% and 22.06%, respectively. In the DFS network, CT + T + Neratinib (N) was the best treatment option with 50.55%, followed by CT + T + P (26.59%) and CT + T + L (20.62%).

**Conclusion:**

This network meta-analysis suggests that dual anti-HER2 blockade with trastuzumab plus either lapatinib or pertuzumab are probably the best treatment options in the (neo)adjuvant setting for HER2-positive breast cancer patients in terms of OS gain. Mature OS results are still expected for the Aphinity trial and for the sequential use of trastuzumab followed by neratinib, the treatment that showed the best performance in terms of DFS in our analysis.

## Introduction

Breast cancer is the most common malignancy among women worldwide accounting for 2.4 million new cases and 523,000 deaths yearly (1). Approximately 15–20% of breast cancers are classified as HER2-positive (HER2+), a subgroup of tumors with a more aggressive clinical phenotype and worse prognosis due to unregulated cell growth and abnormal survival mediated by overexpression of the HER2 protein (2–8).

Currently, there are multiple effective therapies blocking the HER2-pathway in different manners (intra or extracellular). Since the approval of the first HER2-targeted therapy, trastuzumab, in 2001, other anti-HER2-targeted agents have been developed and tested in the metastatic and in the (neo)adjuvant settings, including lapatinib, pertuzumab, neratinib, and TDM1 (9, 10). In the adjuvant setting, trastuzumab was the cornerstone of the anti-HER2 therapy for the treatment of HER2+ breast cancer until recently, when results of T + P and neratinib after T were approved (11–13).

Despite the efficacy of trastuzumab, recurrences do occur and present a serious clinical problem for HER2+ patients since tumors may exhibit *de novo* or acquired resistance (14–20). To overcome trastuzumab resistance, it has been combined with different chemotherapies and other HER2-targeted agents, a strategy known as dual anti-HER2 blockade. Unfortunately, it is not feasible to have adequate pairwise comparative data for all the available treatment options because the number of possible head-to-head comparisons directly expands in a quadratic proportion with the availability of effective agents (21).

Mixed Treatment Comparison (MTC) meta-analysis, a generalization of pairwise meta-analysis, offers an opportunity to a more detailed inference in clinical situations where there are many different treatment options. By combining direct and indirect evidence to compare multiple treatment arms across studies (with the proviso that there is at least one common arm between them), it extends beyond the standard and critical pairwise meta-analysis constraint of only incorporating information from two directly compared arms (21–23).

Within the current context of personalized medicine, where physicians and policy makers must base their decisions on the highest level of available evidence in order to choose one out of multiple available treatment options, the present study aims to summarize the network of evidence supporting the treatment of patients with early and locally advanced HER2+ breast cancer in terms of overall survival (OS) and disease-free survival (DFS).

## Materials and Methods

### Search Strategy

MEDLINE, EMBASE, and Cochrane Central Register of Controlled Trials were searched without any restriction of language or year of publication. Different search algorithms were used for each database. Detailed search strategies are described in the Supplementary Material A. To avoid overlooking the results of recent neoadjuvant and adjuvant trials, we also conducted an electronic search of the main international congress abstracts: the American Society of Clinical Oncology, the San Antonio Breast Cancer Symposium, the European Society for Medical Oncology (ESMO), and the St. Gallen International Breast Cancer Conference. Additionally, other relevant trials were sought by reviewing the reference lists of the selected trials.

### Selection Criteria

The pre-specified eligibility criteria for trials included in this review were as follows: phase II or III randomized controlled trials that compared CT plus any anti-HER2 therapy with CT alone or any different combination of CT plus anti-HER2 therapy in the adjuvant or neoadjuvant settings (criteria for HER2-positivity as standard for HER2 adjuvant and neoadjuvant trials). Trials with two or more treatment arms were considered eligible for this work. Following the GCP (Good Clinical Practice) guidelines, endocrine therapy was offered to patients with hormonal receptor positive tumors as part of their adjuvant treatment in all included trials, but studies in which the main objective was to evaluate different adjuvant endocrine therapies were excluded. Two independent reviewers (Paulo Filho and Caroline Albuquerque) reviewed the list of citations obtained from the literature search and applied the pre-specified eligibility criteria above to selected the articles to be included. A third reviewer (Márcio Debiasi) adjudicated any discordance.

### Data Extraction

For each trial, two independent authors (Paulo Filho and Caroline Albuquerque) extracted the following data: year of publication, sample size, baseline patients’ characteristics, and outcomes [hazard ratios (HRs) for OS and DFS]. The Cochrane Collaboration risk of bias tool was used to assess studies’ quality (24).

### Definition of Treatment Arms

In order to organize the existing treatment options tested in clinical trials in clinically meaningful arms, some general pre-specified criteria were used to gather the treatment arms as follows:

ARM 1.chemotherapy alone^1^
ARM 2.chemotherapy (see text footnote 1) + trastuzumab 12 months
ARM 3.chemotherapy (see text footnote 1) + trastuzumab ≤ 6 months
ARM 4.chemotherapy (see text footnote 1) (taxane + carboplatin) + trastuzumab 12 months
ARM 5.chemotherapy (see text footnote 1) + lapatinib 12 months
ARM 6.chemotherapy (see text footnote 1) + trastuzumab 3 months → lapatinib 9 months (sequential to trastuzumab)
ARM 7.chemotherapy (see text footnote 1) + trastuzumab 12 months + lapatinib^2^ (concomitant with trastuzumab)
ARM 8.chemotherapy (see text footnote 1) + trastuzumab 12 months + pertuzumab (see text footnote 2) (concomitant with trastuzumab)
ARM 9.chemotherapy (see text footnote 1) + trastuzumab 12 months → neratinib 12 months (sequential to trastuzumab)

### Definition of Outcomes

Overall survival was the primary outcome of this study and was similarly defined among studies as the time from randomization until death, using an intention to treat analysis. DFS was defined as time from randomization to death or any DFS event. Definitions of DFS events had slight variations among the trials included and are summarized in the Supplementary Material B.

### Statistical Methods

Network meta-analysis (also commonly referred to as multiple treatment comparison—MTC) is a generalization of classic meta-analysis that combines direct and indirect evidence to compare multiple treatment arms across studies with similar populations and outcomes with the proviso that there is at least one linking-arm between them (22). Direct evidence is defined as the head-to-head comparison between two treatment arms in a clinical trial, while indirect evidence is the estimation of the relative effect between two arms that is obtained indirectly through one or more common comparators. MTC can be regarded as a generalized linear model that can be implemented using one out of two frameworks: Bayesian or frequentist. The Bayesian approach is more commonly used mainly because it is flexible enough to analyze a variety of networks of studies (including multi-arm trials) and yields, for each treatment arm, posterior probabilities estimates that allow ranking the treatment arms in a clinically useful manner (21–23, 25–31). These posterior probabilities are calculated based on the HRs for each comparison and their credibility intervals (CrI).

The trials included in this work reported relative treatment effects as HRs for OS and DFS. These data were pooled in to two separate network meta-analyses, one for each outcome of interest. MTC analyses were carried out using the Bayesian approach and considering both fixed effect model and random effects with homogeneous variability among studies model. Consistency assumption was assessed through posterior plots and the Bayesian *p*-values produced by the node splitting method described by Dias et al. (29). Results were summarized as point estimates and their 95% CrI. HRs were modeled using the software WinBUGS (version 1.4.3).

## Results

**Figure 1:**
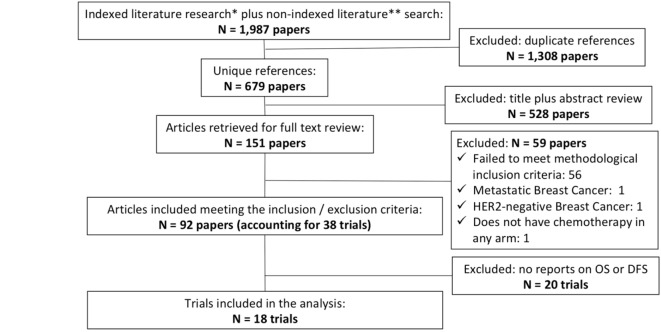
Adapted PRISMA flow diagram. * Indexed literature research: EMBASE, Central (COCHRANE) and PubMed. ** non-indexed literature: *American Society od Cinical Oncology* (ASCO), *San Antonio Breast Cancer Symposium* (SABCS). *European Society for Medical Oncology* (ESMO), St. Gallen International Breast Cancer Conference.

Our electronic search carried out on January 1, 2018 yielded 1,987 unique, of which 92 met the inclusion and exclusion criteria for this review (accounting for 38 trials in total), of which 18 reported OS and/or DFS outcomes. The Figure 1 summarizes the PRISMA (Preferred Reporting Items for Systematic Reviews and Meta-Analysis) chart for the study selection and the supplementary material C describes all the trials included in at least one of the networks (OS and/or DFS). The risk of bias evaluation of each trial was conducted using the Cochrane tool and is described in the supplementary material D (24). All closed loops were considered simple and no inconsistency was found in any of the networks using the “Split Node method” as previously described in the statistical section and the subjective analysis of the direct and indirect evidences reported (Supplementary Material E).

### Overall Survival Network

**Figure 2:**
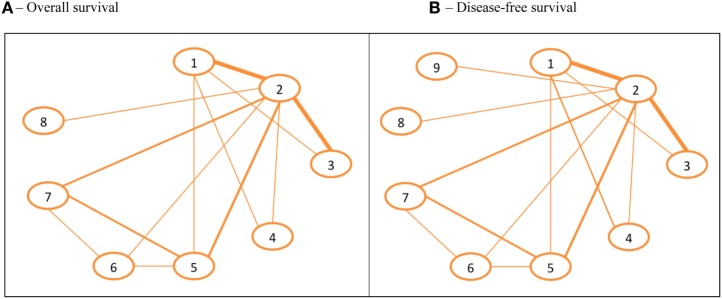
Networks for overall survival (OS) and disease-free survival (DFS). **(A)** OS. **(B)** DFS. The width of the lines represents the relative weight of the direct evidence for a given comparison, based on the number of patients included in trials. ARM 1, chemotherapy alone. ARM 2, chemotherapy + trastuzumab 12 months. ARM 3, chemotherapy + trastuzumab ≤ 6 months. ARM 4, chemotherapy (taxane + carboplatin) + trastuzumab 12 months. ARM 5, chemotherapy + lapatinib 12 months. ARM 6, chemotherapy + trastuzumab 3 months → lapatinib 9 months (sequential to trastuzumab). ARM 7, chemotherapy + trastuzumab 12 months + lapatinib (concomitant with trastuzumab). ARM 8, chemotherapy + trastuzumab 12 months + pertuzumab (concomitant with trastuzumab). ARM 9, chemotherapy + trastuzumab 12 months → NERATINIB 12 months (sequential to trastuzumab).

Fifteen trials published from 2005 to 2017 accounting for 37,837 patients were included in this network, which is described in the Supplementary Material F and in the Figure 2A. The eight treatment arms that constitute this network are ranked in Table 1. Based on the ordered ranks, the regimens containing CT associated with trastuzumab and lapatinib (CT + T + L) for 12 months are probably the best option for OS, with 62.47% of posterior probability of being the best, followed by CT + T + P with 22.06%. On the other hand, arms containing chemotherapy without any anti-HER2-targeted therapy or CT with lapatinib (with no trastuzumab) or CT with trastuzumab for no longer than 6 months are probably the worst options, with posterior probabilities of being the worst of 85, 6.46, and 4.48%, respectively.

**Table 1:** OS and DFS rankings for the (neo)adjuvant treatment strategies available for early and locally advanced HER2 + breast cancer.^a^

	Rank	ARM 1 (%)	ARM 2 (%)	ARM 3 (%)	ARM 4 (%)	ARM 5 (%)	ARM 6 (%)	ARM 7 (%)	ARM 8 (%)	ARM 9 (%)
Disease-free survival	Rank 9	95.88	0.00	1.72	0.65	1.61	0.08	0.00	0.01	0.04
	Rank 8	3.67	0.00	42.29	6.45	47.08	0.32	0.01	0.05	0.13
	Rank 7	0.40	0.39	45.66	11.50	40.31	1.27	0.01	0.22	0.23
	Rank 6	0.05	18.28	9.30	48.34	9.83	11.34	0.48	1.23	1.15
	Rank 5	0.00	56.38	0.88	16.42	0.91	19.11	1.37	2.71	2.20
	Rank 4	0.00	22.80	0.11	10.75	0.24	41.08	7.38	10.89	6.76
	Rank 3	0.00	2.04	0.03	4.13	0.01	18.17	35.39	25.51	14.71
	Rank 2	0.00	0.10	0.00	1.40	0.01	6.75	34.73	32.78	24.23
	Rank 1	0.00	0.01	0.00	0.37	0.00	1.87	20.62	26.59	50.55

Overall survival	Rank 1	0.00	0.35	0.09	3.68	0.03	11.32	62.47	22.06	N/A
	Rank 2	0.01	5.02	0.37	7.98	0.25	35.09	26.76	24.51	N/A
	Rank 3	0.01	26.30	1.28	12.92	1.08	28.63	7.81	21.97	N/A
	Rank 4	0.11	48.01	4.29	15.36	2.87	13.82	2.00	13.53	N/A
	Rank 5	0.43	18.66	19.30	31.87	10.62	8.11	0.76	10.24	N/A
	Rank 6	2.05	1.54	42.90	15.69	31.03	2.26	0.16	4.37	N/A
	Rank 7	12.39	0.12	27.29	9.70	47.65	0.56	0.03	2.26	N/A
	Rank 8	85.00	0.00	4.48	2.80	6.46	0.22	0.00	1.05	N/A

*^a^This table indicates the posterior estimates of the probability of each treatment arm being the best (green squares—rank 1) or the worst (red squares—rank 9 for DFS or rank 8 for OS)*.

*ARM 1, chemotherapy alone. ARM 2, chemotherapy + trastuzumab 12 months. ARM 3, chemotherapy + Trastuzumab ≤ 6 months. ARM 4, chemotherapy (taxane + carboplatin) + trastuzumab 12 months. ARM 5, chemotherapy + lapatinib 12 months. ARM 6, chemotherapy + trastuzumab 3 months → lapatinib 9 months (sequential to trastuzumab). ARM 7, chemotherapy + trastuzumab 12 months + lapatinib (concomitant with trastuzumab). ARM 8, chemotherapy + trastuzumab 12 months + pertuzumab (concomitant with trastuzumab). ARM 9, chemotherapy + trastuzumab 12 months → neratinib 12 months (sequential to trastuzumab)*.

All HRs between treatment arms and their 95% CrI for this network are shown in Table 2. This analysis shows that the combination of CT plus 12 months of dual blockade with trastuzumab and lapatinib almost achieved the significance threshold for superiority when compared to the standard regimen of CT + T for 12 months (HR 0.75; 95% CrI: 0.51–1.01). The comparison between the two dual blockade strategies included in this network (CT + T + L versus CT + T + P) favored the lapatinib arm but was not significant (HR 1.18; 95% CrI: 0.71–2.14).

**Table 2:** Mixed treatment comparison hazard ratios (HRs) and their respective 95% CrI for overall survival and disease-free survival (DFA) comparing the (neo)adjuvant treatment strategies available for early and locally advanced breast cancer.^a,b^

		DFS							
	1	0.62 (0.55–0.72)	0.80 (0.66–0.99)	0.68 (0.51–0.90)	0.81 (0.66–0.98)	0.58 (0.44–0.77)	0.49 (0.37–0.63)	0.49 (0.35–0.66)	0.45 (0.32–0.66)
Overall survival	0.62 (0.52–0.78)	2	1.29 (1.10–1.50)	1.08 (0.82–1.44)	1.30 (1.06–1.55)	0.93 (0.70–1.20)	0.79 (0.61–0.99)	0.78 (0.58–1.02)	0.73 (0.52–1.02)
	0.79 (0.59–1.07)	1.27 (0.99–1.59)	3	0.84 (0.61–1.17)	1.01 (0.78–1.27)	0.73 (0.53–0.97)	0.61 (0.45–0.80)	0.61 (0.43–0.83)	0.57 (0.39–0.82)
	0.68 (0.45–1.04)	1.00 (0.71–1.64)	0.86 (0.53–1.39)	4	1.20 (0.85–1.65)	0.86 (0.58–1.25)	0.73 (0.49–1.04)	0.72 (0.47–1.06)	0.67 (0.43–1.05)
	0.83 (0.61–1.09)	1.34 (0.97–1.69)	1.05 (0.72–1.47)	1.22 (0.74–1.95)	5	0.72 (0.55–0.94)	0.61 (0.48–0.77)	0.60 (0.43–0.84)	0.56 (0.39–0.85)
	0.55 (0.37–0.81)	0.89 (0.59–1.24)	0.70 (0.44–1.06)	0.81 (0.47–1.37)	0.66 (0.47–0.97)	6	0.85 (0.63–1.12)	0.84 (0.57–1.22)	0.78 (0.52–1.22)
	0.47 (0.31–0.66)	0.75 (0.51–1.01)	0.59 (0.38–0.87)	0.69 (0.40–1.13)	0.56 (0.41–0.77)	0.85 (0.57–1.21)	7	0.99 (0.69–1.45)	0.92 (0.62–1.43)
	0.55 (0.35–0.92)	0.89 (0.57–1.38)	0.70 (0.43–1.17)	0.82 (0.45–1.53)	0.67 (0.41–1.16)	1.00 (0.58–1.82)	1.18 (0.71–2.14)	8	0.93 (0.61–1.47)
	—	—	—	—	—	—	—	—	9

*^a^In this table, the diagonal squares numbered 1–9 represent the nine treatment arms included in the OS and/or DFS networks. By crossing the row and the column of two treatment arms in the table, the reader will find the HR for the comparison of these two arms in the corresponding square. HRs were computed considering the hazard of the treatment arm assigned the higher number as the numerator and the hazard of the treatment arm assigned the lower number as the denominator (e.g., when comparing the treatment arms 1 and 2; the reader must consider that it is the hazard of the arm 2 over the hazard of the arm 1)*.

*^b^CrI that do not cross the non-significance threshold (value 1,0) are highlighted in red*.

*ARM 1, chemotherapy alone.^a^ ARM 2, chemotherapy + trastuzumab 12 months. ARM 3, chemotherapy + trastuzumab ≤ 6 months. ARM 4, chemotherapy (taxane + carboplatin) + trastuzumab 12 months. ARM 5, chemotherapy + lapatinib 12 months. ARM 6, chemotherapy + trastuzumab 3 months → lapatinib 9 months (sequential to trastuzumab). ARM 7, chemotherapy + trastuzumab 12 months + lapatinib (concomitant with trastuzumab). ARM 8, chemotherapy + trastuzumab 12 months + pertuzumab (concomitant with trastuzumab). ARM 9, chemotherapy + trastuzumab 12 months → neratinib 12 months (sequential to trastuzumab)*.

Other important findings yielded by this analysis are: the length of trastuzumab treatment probably impacts negatively on OS and the omission of anthracycline did not seem to jeopardize treatment efficacy. Six months of anti-HER2 therapy is probably inferior to 12 months with an estimated 27% increase in the probability of death (HR 1.27; 95% CrI: 0.99–1.59). On the other hand, the schedule composed of a taxane without anthracycline plus trastuzumab for 1 year (TCH—arm 4) had similar efficacy when compared to the standard regimen of CT + T for 12 months (HR 1.00; 95% CrI: 0.71–1.64). It is important to emphasize that more than 90% of patients included in the CT + T for 12 months arm in this comparison did receive an anthracycline-containing regimens.

### DFS Network

This network is composed of 17 trials, published from 2005 to 2017, and includes 40,992 patients. The design of this network as well as the trials included are summarized in Figure 2B and Supplementary Material F, respectively. The ranking of the nine included arms and their HRs are summarized in Tables 1 and 2. Two studies—the ExteNET and the NeoSphere trials—were included only in this network, and not in the OS network, because they only reported DFS data (13, 32, 33). The inclusion of the ExteNET trial to this network brought up that using an additional year of neratinib after the standard chemotherapy plus 1 year of trastuzumab is probably the best treatment option for this outcome, with 50.55% of posterior probability of this arm being the best one (Table 1). Most of the other findings observed at the OS network were confirmed in the DFS network.

## Discussion

It is well established that CT plus trastuzumab is the backbone for the adjuvant and neoadjuvant treatment of the HER2+ early and locally advanced breast cancer patients. This statement is based on a robust body of evidence that comes from several phase III randomized clinical trials and meta-analyses (34). However, uncertainties in the management of HER2+ breast cancer patients remain, such as the optimal chemotherapy regimen that should be administered with trastuzumab and the length of trastuzumab therapy, although the empiric 1-year duration is the standard of care. Recently, the FDA-approved dual blockade with trastuzumab plus pertuzumab as well as 1 year of neratinib after the completion of 1 year trastuzumab (11, 13). Additionally, different strategies to inhibit the HER2-pathway, such as those with pertuzumab, lapatinib, and neratinib, have already been tested creating multiple possible comparisons, which cannot be assessed by classical meta-analysis methodology and have not been tested in proper head-to-head comparisons. The present work is the first to model HRs for time-to-event outcomes (OS and DFS) in unique mathematical models (Mixed Comparison Treatment network meta-analysis—MTC) that include all the available evidence on this matter.

No inconsistencies were found in either of the networks, as presented in the Supplementary Material E. An additional finding that corroborates the robustness of the data presented here is that this analysis identified a HR of 0.62 (95% CrI:0.52–0.78) for the comparison between chemotherapy alone versus chemotherapy plus one year of trastuzumab, which is very close to the results found in a Cochrane meta-analysis (0.66; 95% CI 0.57–0.77) (33). The slightly better results observed in the present analysis can be explained by the fact that all patients in this comparison used 1 year of trastuzumab, while the Cochrane meta-analysis considered together patients that received trastuzumab for 1 year and for less than 6 months (33).

The concept of the dual blockade was proven in this analysis. In the OS network, regimens containing CT plus trastuzumab and lapatinib were probably the best treatment options in this scenario, followed by CT plus trastuzumab and pertuzumab, with posterior probabilities of these regimes being the best of 62.47 and 22.06%, respectively. Questions still remain on this subject, because OS has not yet been published for neratinib and is not yet fully mature for the adjuvant pertuzumab study, the Aphinity Trial. However, these results are robust in showing the benefit of the dual blockade for early breast cancer patients. This finding might seem somehow unexpected given the negative OS results of the ALTTO trial. However, one should note that sample size for this trial was calculated for DFS and the number of events are lower than expected by the inclusion of low risk patients (40% node-negative and 40% small tumors) (555 DFS events instead of the 850 expected events in the first report) (35, 36). The mature OS results of the trials studying trastuzumab plus pertuzumab are expected in the future and may help further supporting dual anti-HER2 blockade in women with curable HER2+ breast cancer.

The positive results of the dual blockade were also confirmed in the DFS network analysis. However, it is important to note that this network counted with an extra arm of sequential neratinib after the standard CT + T from the ExteNET trial and that this arm was probably the best one for this outcome. After this analysis, CT associated with 12 months of trastuzumab might still be the standard treatment for HER2 + early or locally advanced breast cancer patients, though there is a strong suggestion that dual blockade is superior and pertuzumab and neratinib have been approved by the FDA in the adjuvant setting. Despite the negative results of the ALTTO trial, the point estimate in this randomized clinical trial showed benefit and, as discussed before, there might be some conservative bias in the analysis of the results.

## Conclusion

In the era of personalized medicine, HER2-positive breast cancer turned to be a curable disease with multiple effective treatment options creating an urgent need for more comprehensive methods for data analysis and biomarkers to select the right treatment for the right patient. This network meta-analysis suggests that combining trastuzumab with either lapatinib or pertuzumab is probably the best strategy in terms of OS gain in this clinical scenario. However, at this moment, only the combination of trastuzumab and pertuzumab as adjuvant treatment has been FDA approved. Mature OS results are still expected for the Aphinity trial and for the sequential use of trastuzumab followed by neratinib (the treatment strategy that have shown the best results in terms of DFS in our analysis). As evidence-based medicine evolves, analyses from complex networks of trials must be combined with the patient’s individual risk of recurrence, safety profile, and patient’s preferences to support choices in an evidence-based clinical practice.

## Author Contributions

Study conception and design, data interpretation, and manuscript writing and reviewing: MD, CP, PZ, CB, HC, JD, PG, BB, DF, RG, PF, CA, TR, EA, and OO. Literature review: MD, CP, PZ, CB, BB, RG, PF, CA, and EA. Data extraction: MD, PF, and CA. Statistical analysis: MD, PZ, HC, and JD.

## Conflict of Interest Statement

MD, ROCHE: honoraria, LIBBS: honoraria. CP, PFIZER: research funding, NOVARTIS: honoraria. PZ, no conflicts of interest to declare. CB, NOVARTIS, ROCHE/GENENTECH, PFIZER, GSK, SANOFI, BOEHRINGER INGELHEIM, and EISAI: honoraria; BOEHRINGER INGELHEIM, NOVARTIS, ROCHE/GENENTECH, ASTELLAS PHARMA, GSK, EISAI, and PFIZER: consulting or advisory role; PFIZER, NOVARTIS, AMGEN, ASTRAZENECA, BOEHRINGER INGELHEIM, GSK ROCHE/GENENTECH, LILLY, SANOFI, TAIHO PHARMACEUTICAL, MYLAN, MERIMACK, MERCK, ABBVIE, ASTELLAS, BIOMARIN, BMS, DAIICHI SANKYO, ABRAXIS BIOSCIENCE, AB SCIENCE, ASANA BIOSCIENCES, MEDIVATION, EXELIXIS, IMCLONE SYSTEMS, LEO PHARMA, and MILLENIUM: research funding. HC, no conflicts of interest to declare. JD, no conflicts of interest to declare. PG, no conflicts of interest to declare. BB, no conflicts of interest to declare. DF, AMGEN, JANSEN: consulting or advisory role, AMGEN: funding for research, AMGEN: travel and accommodations. RG, ASTRAZENECA, MERCK: honoraria. PF, no conflicts of interest to declare. CA, no conflicts of interest to declare. TR, no conflicts of interest to declare. EA, ROCHE: honoraria; ROCHE: consulting or advisory board; ROCHE: research funding; ROCHE, GSK: travel and accommodations. OO, no conflicts of interest to declare.

## Footnotes

^1^Due to heterogeneity between studies, different chemotherapy backbones were accepted (most of which included anthracyclines and taxanes), with the exception of the arm #4 (from the BCIRG006 trial) in which all patients received chemotherapy with docetaxel + carboplatin.

^2^Lapatinib and pertuzumab in these arms were given from 3 to 12 months concomitantly with trastuzumab.
