# Supplementary material for: Efficacy of Anti-HER2 Agents in Combination With Adjuvant or Neoadjuvant Chemotherapy for Early and Locally Advanced HER2-Positive Breast Cancer Patients: A Network Meta-Analysis
Source: Front Oncol. 2018 May 22;8:156. doi: 10.3389/fonc.2018.00156 (PMC5972314; doi:10.3389/fonc.2018.00156)
Supplement: Supplementary file 1 [file table_1.PDF]

SUPPLEMENTARY MATERIAL

SUPPLEMENTARY MATERIAL A: literature search designed by Marcio Debiasi

Database: MEDLINE (through Pubmed)

Search date: 05/31/2015 (updated on 01/01/2018)

PATIENTS (breast cancer**)**

1. Breast cancer **(354,751 papers)**
2. Breast neoplasms **(297,311 papers)**
3. Breast tumor **(320,397 papers)**
4. Breast tumour **(299,927 papers)**
5. (((Breast cancer) OR breast neoplasms) OR breast tumor) OR breast tumour **(358,534 papers)**

INTERVENTIONS/COMPARISONS (Isolated chemotherapy or chemotherapy associated with any anti-HER2 inhibitor in the adjuvant or neoadjuvant settings**)**

6. Adjuvant treatment **(212,319 papers)**
7. Adjuvant therapy **(192,589 papers)**
8. Adjuvant chemotherapy **(64,844 papers)**
9. Neoadjuvant treatment **(28,677 papers)**
10. Neoadjuvant therapy **(26,521 papers)**
11. Neoadjuvant chemotherapy **(24,137 papers)**
12. (((((Adjuvant treatment) OR Adjuvant therapy) OR Adjuvant chemotherapy) OR Neoadjuvant treatment) OR Neoadjuvant therapy) OR Neoadjuvant chemotherapy **(230,558 papers)**
13. HER2 pathway inhibitors **(648 papers)**
14. Trastuzumab **(9,164 papers)**
15. Herceptin **(9,591 papers)**
16. Lapatinib **(2,253 papers)**
17. Tykerb **(2,255 papers)**
18. Pertuzumab **(728 papers)**
19. Perjeta **(728, papers)**
20. T-DM1 **(298, papers)**
21. Trastuzumab emtansine **(404 papers)**
22. ((((((((HER2 pathway inhibitors) OR trastuzumab) OR herceptin) OR lapatinib) OR tykerb) OR pertuzumab) OR perjeta) OR T-DM1) OR Trastuzumab emtansine **(11,342 papers)**
23. 12 AND 22 **(2,488 papers)**

PATIENTS (breast cancer**)** **PLUS INTERVENTIONS/COMPARISONS (**Isolated chemotherapy or chemotherapy associated with any anti-HER2 inhibitor in the adjuvant or neoadjuvant settings**)**

24. 5 AND 23 **(2.284 papers)**
25. FILTER: CLINICAL TRIALS **(332 papers)**

26. FILTER: HUMANS **(332 papers)**

Database: Cochrane Central Register of Controlled Trials

Search date: 06/01/2015 (updated on 01/01/2018).
http://crso.cochrane.org

PATIENTS (breast cancer)

1. Breast cancer [TI, AB, KY] **(21,528 papers)**
2. Breast neoplasms [TI, AB, KY] **(9,133 papers)**
3. Breast tumor [TI, AB, KY] **(454 papers)**
4. Breast tumour [TI, AB, KY] **(56 papers)**
5. (((Breast cancer [TI, AB, KY]) OR breast neoplasms [TI, AB, KY]) OR breast tumor [TI, AB, KY]) OR breast tumour [TI, AB, KY] **(22,947 papers)**

INTERVENTIONS/COMPARISONS (Isolated chemotherapy or chemotherapy associated with any anti-HER2 inhibitor in the adjuvant or neoadjuvant settings**)**

6. Adjuvant treatment [TI, AB, KY] **(2,381 papers)**
7. Adjuvant therapy [TI, AB, KY] **(5,519 papers)**
8. Adjuvant chemotherapy [TI, AB, KY] **(4,582 papers)**
9. Neoadjuvant treatment [TI, AB, KY] **(463 papers)**
10. Neoadjuvant therapy [TI, AB, KY] **(1,756 papers)**
11. Neoadjuvant chemotherapy [TI, AB, KY] **(1,729 papers)**
12. (((((Adjuvant treatment [TI, AB, KY]) OR Adjuvant therapy [TI, AB, KY]) OR Adjuvant chemotherapy [TI, AB, KY]) OR Neoadjuvant treatment [TI, AB, KY]) OR Neoadjuvant therapy [TI, AB, KY]) OR Neoadjuvant chemotherapy [TI, AB, KY] **(12,097 papers)**
13. HER2 pathway inhibitors [TI, AB, KY] **(0 papers)**
14. Trastuzumab [TI, AB, KY] **(1,446 papers)**
15. Herceptin [TI, AB, KY] **(120 papers)**
16. Lapatinib [TI, AB, KY] **(435 papers)**
17. Tykerb [TI, AB, KY] **(6 papers)**
18. Pertuzumab [TI, AB, KY] **(254 papers)**
19. Perjeta [TI, AB, KY] **(7 papers)**
20. T-DM1 [TI, AB, KY] **(93 papers)**
21. Trastuzumab emtansine [TI, AB, KY] **(130 papers)**
22. ((((((((HER2 pathway inhibitors [TI, AB, KY]) OR trastuzumab [TI, AB, KY]) OR Herceptin [TI, AB, KY]) OR lapatinib [TI, AB, KY]) OR tykerb [TI, AB, KY]) OR pertuzumab [TI, AB, KY]) OR perjeta [TI, AB, KY]) OR T-DM1 [TI, AB, KY]) OR Trastuzumab emtansine [TI, AB, KY] **(1,669 papers)**
23. 12 AND 22 **(540 papers)**

PATIENTS (breast cancer) **PLUS INTERVENTIONS/COMPARISONS (**Isolated chemotherapy or chemotherapy associated with any anti-HER2 inhibitor in the adjuvant or neoadjuvant settings**)**

24. 5 AND 23 **(525 papers)**

Database: EMBASE

Search date: 05/31/2015 (not available for update on 01/01/2018).
http://www.embase.com/;jsessionid=522AD678F786F74155F1CCCF6AADA99C#quickSearch/default

Search for journals

PATIENTS (breast cancer)

1. Breast cancer **(453.995 papers)**
2. Breast neoplasms **(15.612 papers)**
3. Breast tumor **(227.449 papers)**
4. Breast tumour **(25.209 papers)**
5. (((Breast cancer) OR breast neoplasms) OR breast tumor) OR breast tumour **(456.382 papers)**

INTERVENTIONS/COMPARISONS (Isolated chemotherapy or chemotherapy associated with any anti-HER2 inhibitor in the adjuvant or neoadjuvant settings**)**

6. Adjuvant treatment **(115.282 papers)**
7. Adjuvant therapy **(153.136 papers)**
8. Adjuvant chemotherapy **(82.358 papers)**
9. Neoadjuvant treatment **(23.080 papers)**
10. Neoadjuvant therapy **(26.358 papers)**
11. Neoadjuvant chemotherapy **(22.455 papers)**
12. (((((Adjuvant treatment) OR Adjuvant therapy) OR Adjuvant chemotherapy) OR Neoadjuvant treatment) OR Neoadjuvant therapy) OR Neoadjuvant chemotherapy **(179.500 papers)**
13. HER2 pathway inhibitors **(651 papers)**
14. Trastuzumab **(25.856 papers)**
15. Herceptin **(7.739 papers)**
16. Lapatinib **(8.092 papers)**
17. Tykerb **(1.029 papers)**
18. Pertuzumab **(2.137 papers)**
19. Perjeta **(150 papers)**
20. T-DM1 **(346 papers)**
21. Trastuzumab emtansine **(854 papers)**
22. ((((((((HER2 pathway inhibitors) OR trastuzumab) OR herceptin) OR lapatinib) OR tykerb) OR pertuzumab) OR perjeta) OR T-DM1) OR Trastuzumab emtansine **(29.993 papers)**
23. 12 AND 22 **(7.311 papers)**

PATIENTS (breast cancer) **E PLUS INTERVENTIONS/COMPARISONS (**Isolated chemotherapy or chemotherapy associated with any anti-HER2 inhibitor in the adjuvant or neoadjuvant settings**)**

24. 5 AND 23 **(6.488 papers)**
25. Excluding articles from that also appear at MEDLINE **(2.928 papers)**
26. FILTER: CLINICAL TRIALS **(1.229 papers)**
27. FILTER: HUMANS **(1.130 papers)**

EMBASE + CENTRAL (COCHRANE) + PUBMED: 1987 papers, of which 61 were excluded due to duplicated references, resulting 1553 trials

SUPPLEMENTARY MATERIAL B: definition of disease-free / invasive disease-free events

	Local Recurrence	Regional Recurrence	Distant Metastases	Contralateral breast cancer	Other Second Primary Cancer	Death	No information
ALTTO	X	X	X	X	X	X	
APHINITY	X	X	X	X		X	
BCIRG006	X	X	X	X*	X	X	
CALGB40601							X
E-2198	X	X	X	X	X	X	
ExteNET	X	X	X	X		X	
FINHER	X	X	X	X*		X	
HERA	X	X	X	X**	X***	X	
MAVROUDIS / HELENIC	X	X	X	X	X	X	
NEOALTTO	X	X	X	X	X	X	
NEOSPHERE	X	X	X	X		X	
NSABPB31_NCCTGN9831	X	X	X	X	X	X	
PACS 04	X	X	X	X		X	
PHARE	X	X	X	X	X	X	
SHORTHER							X
SOLD							X
TEACH	X	X	X	X	X****	X	

* Invasive disease only
** Includes DCIS but not LCIS
*** Excludes basal-cell or squamous-cell carcinoma of the skin or carcinoma in situ of the cervix
**** Excludes carcinoma of the skin, melanoma in situ or carcinoma in situ of the cervix

SUPPLEMENTARY MATERIAL C: description of the trials included in at least one network (overall survival, disease-free survival or cardiotoxicity)

STUDY	Chemo setting	Industry sponsored	Which industry	Phase	Number of patients	Multicentric	N Countries
ALTTO [40-42]	Adj*	Yes: fully	GSK / Novartis	III	8381	Yes	44
APHINITY [11]	Adj*	Yes: fully	Roche / Genentech	III	4805	Yes	43
BCIRG006 [43-45]	Adj*	Yes: fully	More than one industry	III	3222	Yes	41
CALGB40601 [46-47]	Adj*	Yes: fully	Schering-Plough	II	179	Yes	N/A
E-2198 [48]	Adj*	No	----	II	227	Yes	1
EXTENET [34-35]	Adj*	Yes: fully	Puma Biotech	III	2840	Yes	40
FINHER [49-50]	Adj*	Yes: partially	More than one industry	III	232	Yes	N/A
HERA/BIG01-01 [13, 51-56]	Adj*	Yes: fully	Roche	III	3401	Yes	39
MAVROUDIS [57-58]	Adj*	No	----	N/A	481	Yes	1
NCCTG N9831 [59-65]	Adj*	Yes: partially	Genentech	III	2184	N/A	N/A
NSABP B31 [60-61, 64-67]	Adj*	Yes: partially	Genentech	III	1736	N/A	N/A
NEOALTO [68-71]	Neo**	Yes: fully	GSK	III	455	1	23
NEOSPHERE [72-73]	Neo**	Yes: fully	Roche	II	417	Yes	16
PACS 04 [74]	Adj*	Yes: partially	Roche	III	528	Yes	2
PHARE [75-76]	Adj*	No	----	III	3380	Yes	1
SHORTHER [77-78]	Adj*	No	----	III	1253	Yes	1
SOLD [79]	Adj*	No	----	III	2176	Yes	5
TEACH [80-81]	Adj*	Yes: fully	GSK	III	3147	Yes	33

SUPPLEMENTARY MATERIAL D: risk of bias evaluation of all trials that were included in at least on network (overall survival or disease-free survival)

STUDY	RISK OF BIAS EVALUATION DOMINIUM (21)				
	Allocation sequence	Allocation conceal	Blinding	Incomplete outcome	Selective Reporting
ALTTO	Unclear	Unclear	High risk	Unclear	Unclear
APHINITY	Low risk	Low risk	Low risk	Low risk	Low risk
BCIRG006	Unclear	Unclear	High risk	Low risk	Low risk
CALGB40601	Unclear	Unclear	High risk	Low risk	Low risk
E-2198	Low risk	Low risk	High risk	Low risk	Low risk
EXTENET	Unclear	Unclear	Unclear	Unclear	Unclear
FINHER	Low risk	Low risk	High risk	Low risk	Low risk
HERA/BIG01-01	Unclear	Unclear	High risk	Low risk	Low risk
MAVROUDIS	Low risk	Low risk	High risk	Low risk	Low risk
NCCTG N9831	Unclear	Unclear	High risk	Low risk	Low risk
NEOALTO	Low risk	Low risk	High risk	Low risk	Low risk
NEOSPHERE	Low risk	Low risk	High risk	Low risk	Low risk
NSABP B31	Unclear	Unclear	High risk	Low risk	Low risk
PACS 04	Unclear	Unclear	High risk	Low risk	Low risk
PHARE	Low risk	Low risk	High risk	Low risk	Low risk
SHORTHER	Unclear	Unclear	High risk	Low risk	Low risk
SOLD	Unclear	Unclear	High risk	Low risk	Low risk
TEACH	Low risk	Low risk	Low risk	Low risk	Low risk

<u>SUPPLEMENTARY MATERIAL E</u>: Inconsistency analysis – direct and indirect evidences and Bayesian p-values for those comparisons that have both evidences available from a closed loop composed of more than two studies.

TREATMENT COMPARISON	OUTCOME	DIRECT EVIDENCE – PAIRWISE COMPARISON	DIRECT EVIDENCE - MTC	INDIRECT EVIDENCE - MTC	MTC SUMMARY: DIRECT + INDIRECT EVIDENCES	INCONSISTENCY SPLIT NODE: P-VALUE
**2 - 1**	**OS**	0.62 (0.50-0.77)	0.61 (0.50-0.80)	0.69 (0.42-1.08)	0.62 (0.52-0.78)	0.60
	**DFS**	0.67 (0.60-0.75)	0.63 (0.54-0.76)	0.61 (0.43-0.85)	0.62 (0.55-0.72)	0.86
**3 - 1**	**OS**	0.55 (0.27-1.12)	0.55 (0.25-1.19)	0.83 (0.61-1.21)	0.79 (0.59-1.07)	0.32
	**DFS**	0.65 (0.38-1.12)	0.65 (0.36-1.16)	0.82 (0.67-1.04)	0.80 (0.66-0.99)	0.45
**5 - 1**	**OS**	0.99 (0.74-1.32)	0.99 (0.63-1.54)	0.72 (0.47-1.03)	0.83 (0.61-1.09)	0.23
	**DFS**	0.83 (0.69-0.99)	0.83 (0.60-1.14)	0.79 (0.58-1.04)	0.81 (0.66-0.98)	0.78
**3 - 2**	**OS**	1.31 (1.09-1.59)	1.31 (1.02-1.69)	0.87 (0.38-1.93)	1.27 (0.99-1.59)	0.32
	**DFS**	1.30 (1.16-1.46)	1.30 (1.11-1.55)	1.02 (0.55-1.86)	1.29 (1.10-1.50)	0.44
**5 - 2**	**OS**	1.18 (0.85-1.62)	1.21 (0.81-1.61)	1.67 (0.97-2.71)	1.34 (0.97-1.69)	0.24
	**DFS**	1.30 (1.11-1.52)	1.27 (0.97-1.62)	1.34 (0.93-1.92)	1.30 (1.06-1.55)	0.79

SUPPLEMENTARY MATERIAL F: Network for overall survival

STUDY	COMPARISON BETWEEN ARMS	HR	COMPARISON BETWEEN ARMS	HR	COMPARISON BETWEEN ARMS	HR
BCIRG006	2 vs. 1	0.59 (0.42-0.85)	4 vs. 1	0.66 (0.47-0.93)	----------	--
HERA	2 vs. 1	0.66 (0.47-0.91)	----------	--	----------	--
NSABP_B31 AND NCCTGN9831	2 vs. 1	0.61 (0.50-0.75)	----------	--	----------	--
PACS 04	2 vs. 1	1.27 (0.68-2.38)	----------	--	----------	--
FINHER	3 vs. 1	0.41 (0.16-1.08)	----------	--	----------	--
TEACH	5 vs. 1	0.99 (0.74-1.31)	----------	--	----------	--
E2198	3 vs. 2	1.37 (0.46-3.13)	----------	--	----------	--
MAVROUDIS	3 vs. 2	1.45 (0.57-3.67)	----------	--	----------	--
PHARE	3 vs. 2	1.46 (1.06-2.01)	----------	--	----------	--
SHORTHER	3 vs. 2					
SOLD	3 vs. 2					
ALTTO	5 vs. 2	1.36 (1.09-1.72)	6 vs. 2	0.91 (0.71-1.16)	7 vs. 2	0.80 (0.62-1.03)
CALGB40601	5 vs. 2	0.82 (0.32-2.06)	7 vs. 2	0.22 (0.06-0.76)	----------	--
NEOALTTO	5 vs. 2	0.86 (0.45-1.63)	7 vs. 2	0.62 (0.30-1.25)	----------	--
APHINITY	8 vs. 2	0.89 (0.66-1.21)	----------	--	----------	--

Legend for treatment arms

ARM 1. CHEMOTHERAPY* ALONE*
ARM 2. CHEMOTHERAPY* + TRASTUZUMAB 12 months
ARM 3. CHEMOTHERAPY * + TRASTUZUMAB ≤6 months
ARM 4. CHEMOTHERAPY (TAXANE + CARBOPLATIN) + TRASTUZUMAB 12 months
ARM 5. CHEMOTHERAPY* + LAPATINIB 12months
ARM 6. CHEMOTHERAPY* + TRASTUZUMAB 3 months → LAPATINIB 9months (sequential to trastuzumab)
ARM 7. CHEMOTHERAPY* + TRASTUZUMAB 12 months + LAPATINIB (concomitant with trastuzumab)
ARM 8. CHEMOTHERAPY* + TRASTUZUMAB 12 months + PERTUZUMAB (concomitant with trastuzumab)

SUPPLEMENTARY MATERIAL G: Network for disease-free survival

STUDY	COMPARISON BETWEEN ARMS	HR	COMPARISON BETWEEN ARMS	HR	COMPARISON BETWEEN ARMS	HR
BCIRG006	2 vs. 1	0.61 (0.48-0.76)	4 vs. 1	0.67 (0.54-0.83)	----------	--
HERA	2 vs. 1	0.64 (0.54-0.76)	----------	--	----------	--
NSABP_B31 AND NCCTGN9831	2 vs. 1	0.52 (0.45-0.60)	----------	--	----------	--
PACS 04	2 vs. 1	0.86 (0.61-1.22)	----------	--	----------	--
FINHER	3 vs. 1	0.42 (0.21-0.83)	----------	--	----------	--
TEACH	5 vs. 1	0.83 (0.70-1.00)	----------	--	----------	--
E2198	3 vs. 2	1.31 (0.79-2.12)	----------	--	----------	--
MAVROUDIS	3 vs. 2	1.58 (0.86-2.10)	----------	--	----------	--
PHARE	3 vs. 2	1.28 (1.05-1.56)	----------	--	----------	--
SHORTHER	3 vs. 2	1.15 (0.91-1.46)	----------	--	----------	--
SOLD	3 vs. 2	1.39 (1.12-1.72)	----------	--	----------	--
ALTTO	5 vs. 2	1.34 (1.13-1.60)	6 vs.2	0.96 (0.80-1.15)	7 vs. 2	0.84 (0.70-1.02)
CALGB40601	5 vs. 2	1.24 (0.61-2.51)	7 vs. 2	0.35 (0.15-0.83)	----------	--
NEOALTTO	5 vs. 2	1.06 (0.66-1.65)	7 vs. 2	0.78 (0.47-1.28)	----------	--
NEOSPHERE	8 vs. 2	0.60 (0.28-1.27)	----------	--	----------	--
APHINITY	8 vs. 2	0.81 (0.66-1.00)	----------	--	----------	--
EXTENET	9 vs. 2	0.67 (0.50-0.91)	----------	--	----------	--

Legend for treatment arms

ARM 1. CHEMOTHERAPY ALONE*
ARM 2. CHEMOTHERAPY + TRASTUZUMAB 12 months
ARM 3. CHEMOTHERAPY + TRASTUZUMAB ≤6 months
ARM 4. CHEMOTHERAPY (TAXANE + CARBOPLATIN) + TRASTUZUMAB 12 months
ARM 5. CHEMOTHERAPY + LAPATINIB 12months
ARM 6. CHEMOTHERAPY + TRASTUZUMAB 3 months → LAPATINIB 9months (sequential to trastuzumab)
ARM 7. CHEMOTHERAPY + TRASTUZUMAB 12 months + LAPATINIB (concomitant with trastuzumab)
ARM 8. CHEMOTHERAPY + TRASTUZUMAB 12 months + PERTUZUMAB (concomitant with trastuzumab)
ARM 9. CHEMOTHERAPY + TRASTUZUMAB 12 months → NERATINIB 12 months (sequential to trastuzumab)
